# Influence of cytochrome P450 2D6 polymorphism on hippocampal white matter and treatment response in schizophrenia

**DOI:** 10.1038/s41537-020-00134-z

**Published:** 2021-01-29

**Authors:** Wonsuk Shin, Minji Bang, Anhye Kim, Doo-Yeoun Cho, Sang-Hyuk Lee

**Affiliations:** 1 Department of Clinical Pharmacology and Therapeutics, CHA Bundang Medical Center, CHA University School of Medicine, Seongnam, Republic of Korea; 2 Department of Psychiatry, CHA Bundang Medical Center, CHA University School of Medicine, Seongnam, Republic of Korea

**Keywords:** Genetics of the nervous system, Schizophrenia, Schizophrenia, Molecular neuroscience

## Abstract

Cytochrome P450 2D6 (CYP2D6) is expressed at high levels in the brain and plays a considerable role in the biotransformation and neurotransmission of dopamine. This raises the question of whether CYP2D6 variations and its impact on the brain can confer susceptibility to schizophrenia. We investigated the possible links among the CYP2D6 genotype, white matter (WM) integrity of the hippocampus, and the treatment response to antipsychotic drugs in Korean patients with schizophrenia (*n* = 106). Brain magnetic resonance imaging and genotyping for CYP2D6 were conducted at baseline. The severity of clinical symptoms and the treatment response were assessed using the Positive and Negative Syndrome Scale (PANSS). After genotyping, 43 participants were classified as intermediate metabolizers (IM), and the remainder (*n* = 63) were classified as extensive metabolizers (EM). IM participants showed significantly higher fractional anisotropy (FA) values in the right hippocampus compared to EM participants. Radial diffusivity (RD) values were significantly lower in the overlapping region of the right hippocampus in the IM group than in the EM group. After 4 weeks of antipsychotic treatment, the EM group showed more improvements in positive symptoms than the IM group. FAs and RDs in the CYP2D6-associated hippocampal WM region were significantly correlated with a reduction in the positive symptom subscale of the PANSS. Greater improvements in positive symptoms were negatively associated with FAs, and positively associated with RDs in the right hippocampal region. The findings suggest that CYP26D-associated hippocampal WM alterations could be a possible endophenotype for schizophrenia that accounts for individual differences in clinical features and treatment responses.

## Introduction

Cytochrome P450 2D6 (CYP2D6), a member of the cytochrome P450 mixed-function oxidase system, is one of the most popular enzymes involved in the metabolism of numerous drugs used in clinical practice. The CYP2D6 gene is highly polymorphic with allelic variants causing a large variability in enzymatic activity. Based on what alleles are carried, individuals are classified as poor (PM), intermediate (IM), extensive (EM), and ultrarapid (UM) metabolizers^1^. Recent evidence shows that CYP2D6 is expressed at high levels in neuronal cells of the brain^2,3^, and plays a considerable role in the biotransformation and neurotransmission of central dopamine^4^. This raises the question of whether individual variations in CYP2D6 can confer susceptibility to schizophrenia^5^.

CYP2D6 in the brain is colocated with the dopamine transporter on membranes of dopaminergic neurons^6^. CYP2D6 and the dopamine transporter are functionally similar because they share a common substrate—[^3^H]GBR-12935—which labels the dopamine transporter complex^7–9^. CYP2D6 also has strong activity for the conversion of dopamine from tyramine that exists in the brain^10^, suggesting its intertwining effects on the central dopaminergic system. Previous studies revealed that CYP2D6 activity is associated with conditions, in which dopamine signaling plays a substantial role in pathophysiology, including Parkinson’s disease^11^, smoking behavior^12^, sexual dysfunction^13^, and suicide^14^. In patients with schizophrenia, although some genetic studies produced negative results^15–17^, LLerena and colleagues reported that the frequency of PMs was significantly lower in patients than in healthy controls^18^. However, the relationship between CYP2D6 and schizophrenia is not conclusive in that the exact role of CYP2D6 in the brain, and dopaminergic system remains poorly understood.

The central expression of CYP2D6 has been found in several brain regions, particularly in the cornu ammonis 1–3 of the hippocampus^2,3^. Hippocampal hyperactivity, which leads to the increased dopamine neuron firing and dopamine hyperresponsiveness, is one of the core mechanisms underlying the pathogenesis of schizophrenia^19,20^. Central CYP2D6 may have an additive effect on alterations in the dopamine concentration in the hippocampus by being involved in dopamine transmission and metabolism at the synapse. In this case, given that perturbations in dopamine signaling are associated with direct changes in white matter (WM) myelination^8,21,22^, it could be assumed that individual variations in CYP2D6 activity in patients with schizophrenia are further reflected in measures of WM integrity. Moreover, inter-patient differences in treatment response to antipsychotic drugs (APDs) could be affected by local cerebral drug metabolism by CYP2D6; although, its level in the brain is very low compared to that in the liver^6,12^. Since the current treatment of schizophrenia relies on APDs that block mesolimbic postsynaptic dopamine transmission^23^, CYP2D6 variability and its associated WM changes may be related to the treatment response of APDs in patients with schizophrenia.

Ethnicity differences are particularly important in understanding the impact of CYP2D6 polymorphism on schizophrenia^24^. About 6–10% of Caucasians are reported as PM, and over 25% of Ethiopians are reported as UM^25^. However, in the Republic of Korea, the frequencies of PM and UM are 0.25% and 1.25%, respectively^26–28^. Most of the CYP2D6 activity is expected in EM (64.25%) and IM (34.25%). CYP2D6*5 is the most common nonfunctional allele (6.13%), and CYP2D6*10 is the most common allele for decreased function (45.00%). The allele frequency of CYP2D6 varies among racial/ethnic groups; in particular, EM and IM alleles are most frequent in the Republic of Korea. Hence, exploring the clinical differences between EM and IM in the Korean population is of great significance.

We investigated the possible links among the CYP2D6 genotype, WM integrity of the hippocampus, and the treatment response to APDs in Korean patients with schizophrenia. We hypothesized that (1) hippocampal WM integrity, measured using diffusion tensor imaging (DTI), would differ between IM and EM patients with schizophrenia; (2) IM and EM patients would show different treatment response to APDs; and (3) CYP2D6-associated WM changes would be associated with treatment response to APDs.

## Results

### Genotyping, sociodemographic, and clinical characteristics of the study participants

The genotype distributions of CYP2D6*5 and CYP2D6*10 in the entire group of participants with schizophrenia were in accordance with the Hardy–Weinberg equilibrium (CYP2D6*5, −/−: *n* = 93, −/del: *n* = 13, del/del: *n* = 0, *χ*^2^ = 0.45, *p* = 0.500; CYP2D6*10, CC: *n* = 24, CT: *n* = 39, TT: *n* = 30, single allele: *n* = 13, *χ*^2^ = 2.31, *p* = 0.129). Forty-three participants were categorized as IMs (activity score [AS] 0.5: *n* = 6; AS 1.0: *n* = 37), and the remaining 63 participants as EMs (AS 1.5: *n* = 39; AS 2.0: *n* = 24). Among the 65 participants who were followed-up for the treatment response after 4 weeks, the genotype distributions of CYP2D6*5 and CYP2D6*10 were also in accordance with the Hardy–Weinberg equilibrium (CYP2D6*5, −/−: *n* = 58, −/del: *n* = 7, del/del: *n* = 0, *χ*^2^ = 0.21, *p* = 0.646; CYP2D6*10, CC: *n* = 15, CT: *n* = 25, TT: *n* = 18, single allele: *n* = 7, *χ*^2^ = 1.07, *p* = 0.302). Twenty five participants belonged to the IM group (AS 0.5: *n* = 4; AS 1.0: *n* = 21), and the others (*n* = 40) to the EM group (AS 1.5: *n* = 25; AS 2.0: *n* = 15). The frequency of IM and EM participants in this study was substantially the same as that reported in a previous study on CYP2D6 genotypes in the Korean population^26^.

Table 1 summarizes the sociodemographic and clinical characteristics of all study participants at baseline. The mean AS was nearly twofold higher in the EM group than in the IM group. There were no significant differences in other baseline characteristics between the IM and EM groups. Eighty five out of 106 participants (80.2%) were recent-onset patients who had developed psychotic symptoms within 5 years. Eighty six participants (81.1%) were APD-naive and the remainder were free of APDs at least for 6 months at the time of enrollment. Neuroimaging data were acquired within 2 weeks after the initiation of APD treatment, if participants were not cooperative because of exacerbated psychotic symptoms (mean duration of APD treatment before magnetic resonance imaging [MRI] scan: 4.4 ± 4.2 days).

**Table 1:** Sociodemographic and clinical characteristics of all subjects at baseline scan.

	IM (*n* = 43)	EM (*n* = 63)	Statistics	*p* Value
Sex (*n*, male/female)	14/29	21/42	*χ*^2^ = 0.007	0.934
Age (years, mean ± SD)	37.9 ± 13.5	35.5 ± 10.9	*t* = 0.980	0.330^d^
ICV (ml, mean ± SD)	1453 ± 160	1465 ± 148	*t* = −0.421	0.675
Duration of illness (months, mean ± SD)	47.3 ± 80.5	32.7 ± 60.9	*t* = 1.06	0.290
Duration of untreated psychosis (months, mean ± SD)	13.0 ± 32.0	15.8 ± 31.1	*t* = −0.440	0.661
Duration of APD before scan (days, mean ± SD)	4.8 ± 4.9	4.3 ± 4.4	*t* = 0.815	0.418^d^
APD dose at scan (mg/day, mean ± SD)^a^	542.3 ± 281.4	528.9 ± 227.6	*t* = 0.269	0.789
Maintenance APD			*χ*^2^ = 4.243	0.515
Paliperidone (*n*)	16	27		
Risperidone (*n*)	11	15		
Amisulpride (*n*)	9	16		
Olanzapine (*n*)	4	3		
Clozapine (*n*)	2	0		
Aripiprazole (*n*)	1	2		
Other psychotropic medication				
Mood stabilizer (*n*)^b^	4	3	*χ*^2^ = 0.854	0.355
Antidepressant (*n*)^c^	5	4	*χ*^2^ = 0.916	0.338
2D6 activity score (mean ± SD)	0.93 ± 0.18	1.69 ± 0.24	*t* = −18.628	<0.001
PANSS (mean ± SD)				
Positive symptom	29.3 ± 6.2	29.9 ± 8.2	*t* = −0.381	0.704
Negative symptom	28.3 ± 7.6	25.4 ± 9.7	*t* = 1.607	0.111
General psychopathology	60.1 ± 14.6	54.9 ± 14.8	*t* = 1.805	0.074

*IM* intermediate metabolizer, *EM* extensive metabolizer, *SD* standard deviation, *ICV* intracranial volume, *APD* antipsychotic drug, *PANSS* Positive and Negative Syndrome Scale.

^a^APD doses were converted to the equivalent of chlorpromazine.

^b^All subjects receiving mood stabilizer were taking divalproex sodium.

^c^Antidepressants administered by subjects were escitalopram (*n* = 4), sertraline (*n* = 1), paroxetine (*n* = 3), and duloxetine (*n* = 1).

^d^Levene’s test for equality of variances indicated that the variances did not assumed to be equal between the two groups.

### Baseline comparison of hippocampal WM connectivity between the IM and EM groups

**Fig. 1:**
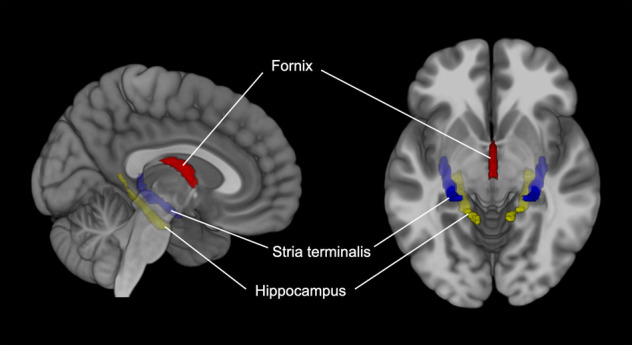
White matter regions of interest. Hippocampal WM regions (hippocampal cingulum, fornix, and stria terminalis) were chosen as ROIsbased on the JHU DTI-based probabilistic tractography atlas.

**Fig. 2:**
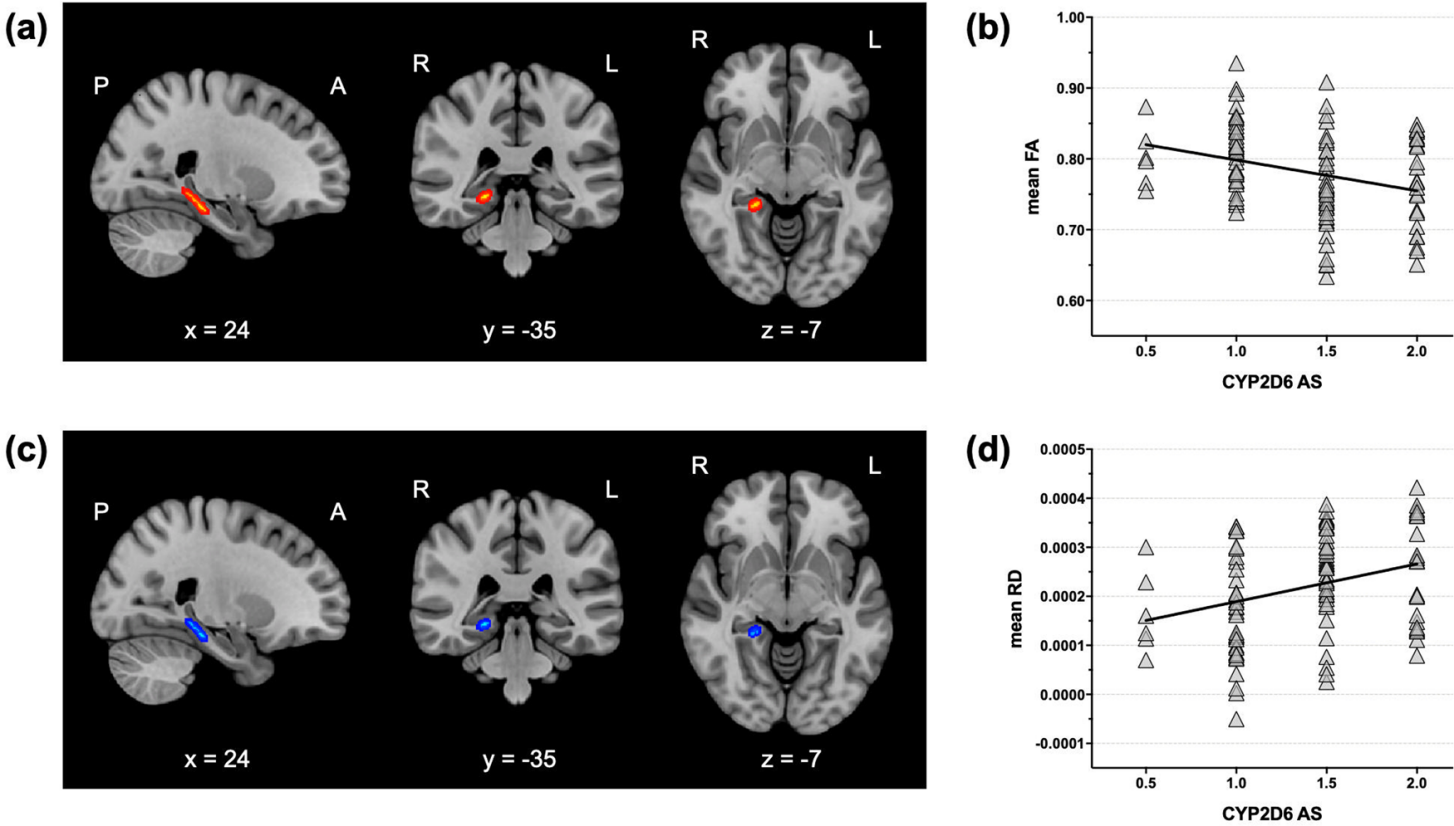
Comparison of hippocampal white matter connectivity between IM and EM patients with schizophrenia. The IM group showed significantly increased FAs (**a**) and decreased RDs (**c**) compared to the EM group in the right hippocampal white matter region (TFCE-corrected *p* < 0.05). The mean FAs (**b**) and RDs (**d**) in this region were significantly correlated with the activity score of CYP2D6 in participants with schizophrenia. IM intermediate metabolizer, EM extensive metabolizer, FA fractional anisotropy, RD radial diffusivity, TFCE threshold-free cluster enhancement.

For neuroimaging analysis, hippocampal WM regions were defined as regions of interest (ROIs) based on the Johns Hopkins University (JHU) DTI-based probabilistic tractography atlas (Fig. 1)^29^. Fractional anisotropy (FA) values were higher in the right hippocampus in the IM group than in the EM group (Fig. 2a,b). Radial diffusivity (RD) values were significantly lower in the overlapping region of the hippocampus in the IM group as compared to the EM group (Fig. 2c, d). The result was not changed after controlling for sex, age, intracranial volume (ICV), duration of illness, and APD dose at scan as covariates. No significant differences were observed for axial (AD) and mean (MD) diffusivity between the two groups.

### Relationships among CYP2D6 activity, hippocampal WM connectivity, and the treatment response

Baseline characteristics between the IM and EM groups of 4-week follow-up participants were not significantly different except for the severity of general psychopathology (Table 2). Both groups were significantly improved across all symptom domains after 4 weeks of APD treatment (IM: paired-*t* > 11.651, *p* < 0.001; EM: paired-*t* > 11.957, *p* < 0.001). EM patients showed a trend of less-severe clinical symptoms than IM patients at the 4-week follow-up. When the percentages of reduction in the Positive and Negative Syndrome Scale (PANSS)^30^ subscale scores were compared, the EM group showed significantly greater improvements in positive symptoms compared to the IM group.

**Table 2:** Sociodemographic and clinical characteristics of the 4-week follow-up group.

	IM (*n* = 25)	EM (*n* = 40)	Statistics	*p* Value
Sex (*n*, male/female)	9/16	12/28	*χ*^2^ = 0.253	0.615
Age (years, mean ± SD)	40.1 ± 13.3	34.7 ± 11.8	*t* = 1.711	0.092
ICV (ml, mean ± SD)	1458 ± 167	1468 ± 154	*t* = −0.239	0.812
Duration of illness (months, mean ± SD)	47.6 ± 85.2	26.6 ± 54.5	*t* = *1.100*	0.279^e^
Duration of untreated psychosis (months, mean ± SD)	12.3 ± 24.0	15.5 ± 31.2	*t* = *−0.431*	0.668
Duration of APD before scan (days, mean ± SD)	5.3 ± 5.1	3.7 ± 3.3	*t* = 1.341	0.188^e^
APD dose at scan (mg/day, mean ± SD)^a^	654.5 ± 293.5	528.7 ± 214.2	*t* = 1.993	0.051
APD dose after 4 weeks (mg/day, mean ± SD)^b^	789.6 ± 276.3	754.5 ± 273.2	*t* = 0.501	0.618
Maintenance APD			*χ*^2^ = 5.020	0.285
Paliperidone (*n*)	11	21		
Risperidone (*n*)	2	6		
Amisulpride (*n*)	7	11		
Olanzapine (*n*)	3	2		
Clozapine (*n*)	2	0		
Aripiprazole (*n*)	0	0		
Other psychotropic medication				
Mood stabilizer (*n*)^c^	4	7	*χ*^2^ = 0.025	0.875
Antidepressant (*n*)^d^	2	5	*χ*^2^ = 0.324	0.569
2D6 activity score (mean ± SD)	0.92 ± 0.19	1.69 ± 0.25	*t* = −14.246	<0.001
Baseline PANSS (mean ± SD)				
Positive symptom	31.1 ± 4.8	31.2 ± 8.7	*t* = −0.042	0.967^f^
Negative symptom	30.6 ± 6.8	26.5 ± 9.9	*t* = 1.815	0.074
General psychopathology	65.0 ± 12.1	56.8 ± 15.1	*t* = 2.303	0.025
PANSS after 4 weeks (mean ± SD)				
Positive symptom	19.7 ± 6.1	16.6 ± 6.2	*t* = 1.975	0.053
Negative symptom	16.0 ± 6.5	12.9 ± 5.3	*t* = 2.071	0.042
General psychopathology	47.0 ± 12.1	37.6 ± 11.8	*t* = 3.106	0.003
% Reduction in the severity of clinical symptoms (mean ± SD)^e^				
Positive symptom	36.6 ± 16.4	47.2 ± 13.7	*t* = −2.826	0.006
Negative symptom	48.3 ± 15.7	48.6 ± 18.6	*t* = −0.069	0.946
General psychopathology	28.0 ± 11.2	32.6 ± 18.2	*t* = −1.134	0.261

IM intermediate metabolizer, EM extensive metabolizer, SD standard deviation, ICV intracranial volume, APD antipsychotic drug, PANSS Positive and Negative Syndrome Scale.

^a^APD doses were converted to the equivalent of chlorpromazine.

^b^Two subjects were taking lithium, and the others (*n* = 9) were taking divalproex.

^c^Antidepressants administered by subjects were escitalopram (*n* = 4), paroxetine (*n* = 2), and bupropion (*n* = 1).

^d^% reduction In the PANSS scores = ([Baseline–4-week follow-up]/Baseline) × 100.

^e^Levene’s test for equality of variances indicated that the variances did not assumed to be equal between the two groups.

**Fig. 3:**
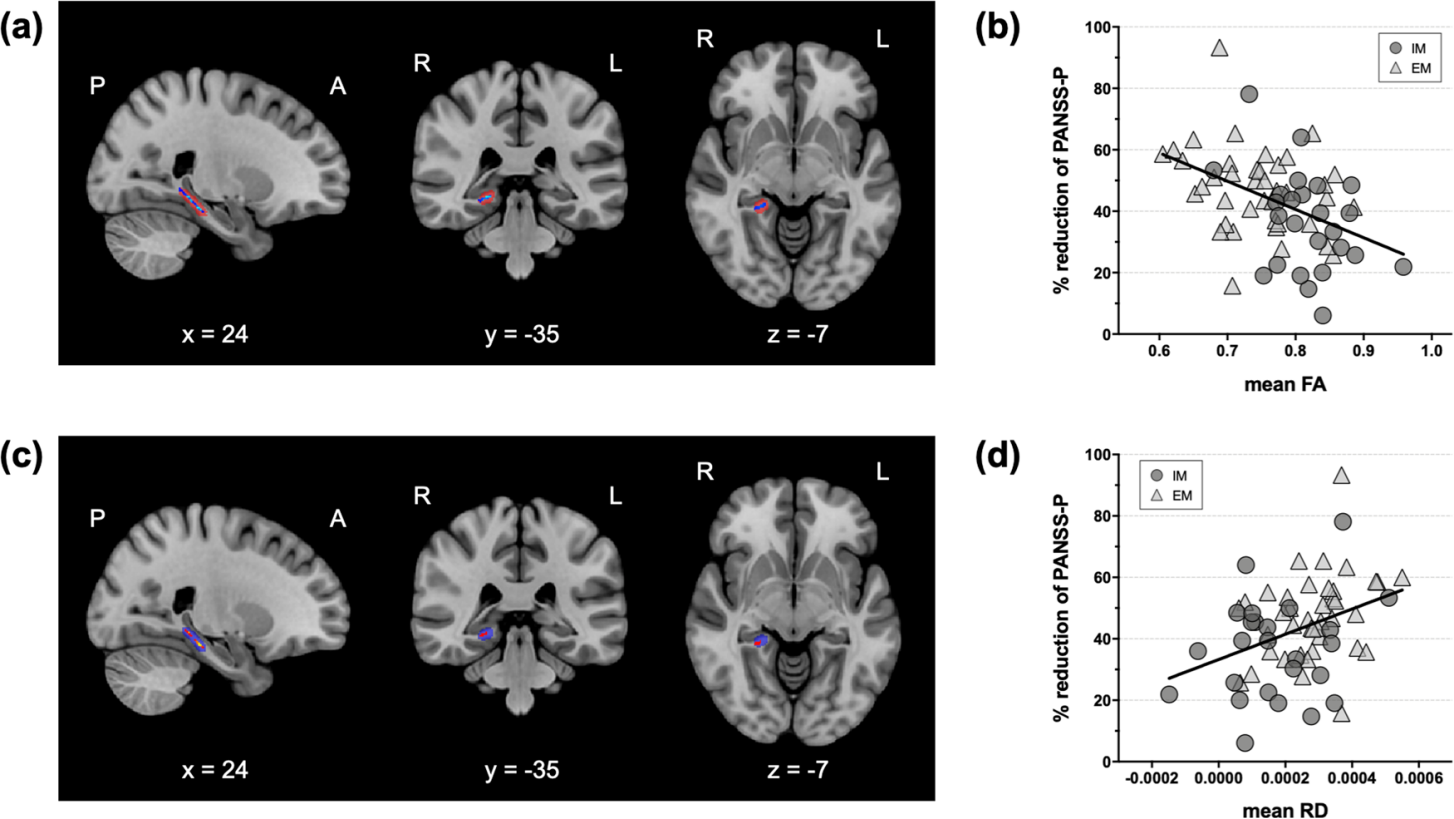
Associations between CYP2D6-associated hippocampal white matter connectivity and treatment response after 4 weeks of APD treatment. Higher FAs (**a**, **b**) and lower RDs (**c**, **d**) were associated with lesser improvements in positive symptoms (TFCE-corrected *p* < 0.05). The red (**a**) and blue areas (**b**) indicate the regions showing significant differences in FAs and RDs between the IM and EM groups, respectively. CYP2D6 cytochrome P450 2D6, APD antipsychotic drug, FA fractional anisotropy, RD radial diffusivity, PANSS-P the positive symptom subscale of the Positive and Negative Syndrome Scale, TFCE threshold-free cluster enhancement, IM intermediate metabolizer, EM extensive metabolizer.

As shown in Fig. 3, voxel-wise correlation analysis in the CYP2D6-associated WM region of the right hippocampus revealed that FAs and RDs in this region were significantly associated with the percentages of reduction in positive symptoms. The percentages of reduction were negatively correlated with FAs (Fig. 3a, b) and positively correlated with RDs (Fig. 3c,d) in the right hippocampal region.

## Discussion

This study examined the genetic influence of CYP2D6 polymorphism on hippocampal WM connectivity and treatment response in patients with schizophrenia. We demonstrated that IM patients had significantly higher FA and lower RD values in the right hippocampal WM region compared to EM patients. In the entire group of patients with schizophrenia, the CYP2D6 ASs were associated with the FA and RD values of the right hippocampal WM region—negatively with the FA and positively with the RD values. After 4 weeks of APD treatment, the EM group showed a significantly higher percentage of reduction in the severity of positive symptoms compared to the IM group. In accordance with aforementioned findings, higher FA and lower RD values in the CYP2D6 activity-associated WM cluster were significantly correlated with the less-improved percentages of reduction in the PANSS positive symptom subscale scores. The present results suggest that CYP2D6 polymorphism may affect structural alterations in WM connectivity of the brain underlying the pathogenesis and clinical manifestation of schizophrenia.

A lack of neuroimaging studies had investigated the impact of CYP2D6 polymorphism on the brain. A previous study with heathy individuals of central European origin showed that lower CYP2D6 activity was associated with higher levels in resting brain perfusion in the thalamus and hippocampus^31^. Elevated regional cerebral blood flow in the hippocampus provides supporting evidence for hippocampal hyperactivity in patients with schizophrenia^32–34^. Excessive hippocampal activity, which is mediated by the dysfunction of hippocampal gamma-aminobutyric acid interneurons^35^, causes a overdrive of the mesolimbic dopamine system that leads to the development of schizophrenia^36,37^. Such neuronal stimulation induces adaptive myelination, resulting in an increase in myelin thickness and integrity of the downward WM pathways^38^. Hence, we carefully assume that our finding of higher hippocampal WM connectivity in IMs with schizophrenia may reflect that the low activity of CYP2D6 is one contributing factor to hippocampal hyperactivity and dopamine dysregulation.

After 4 weeks of APD treatment, participants with schizophrenia who had extensive CYP2D6 activity showed a better improvement in positive symptoms than those with intermediate activity. From the pharmacological point of view, the response to APDs is correlated with their plasma concentration in most cases^39–41^. In this study, the most frequently administered APDs in the follow-up group were paliperidone and amisulpride, which are mainly excreted by the kidney and are not vastly influenced by cytochrome P450 (refs. ^42,43^). Since there was no significant between-group difference in maintenance APD doses, the exposure to APDs might not differ between the IM and EM groups. Therefore, our finding of different treatment response according to CYP2D6 activity may be owing to the ripple effect of CYP2D6 on central dopamine signaling, not the local cerebral metabolism of APDs.

We further demonstrated that higher FAs and lower RDs, correlated with decreased CYP2D6 ASs, were associated with poor treatment response in positive symptoms in participants with schizophrenia. At first glance, this finding seems inconsistent with previous studies that suggested that decreased WM connectivity was a predictive marker for poor treatment outcome^44,45^. However, there are also alternative results showing that increased fronto-temporal WM connectivity at baseline was negatively associated with severity reduction in positive symptoms in patients with schizophrenia^46,47^. The discrepancy among these findings may partly be related to heterogeneity in study populations. A recent DTI study using a fully data-driven approach revealed distinct patterns of WM abnormalities in APD-naive, first-episode patients with schizophrenia, suggesting neurobiological variability in the diagnostic category of schizophrenia^48^. Another possible speculation may involve a moderating role of CYP2D6 activity in the regulation of the central dopamine system and WM connectivity. As indicated by our findings, decreased activity of CYP2D6 seems to contribute to hyperactivity of the hippocampal dopamine system by enhancing hippocampal hyperactivity. This change could result in increased local redundant WM tracts^49,50^. Hence, higher FAs and lower RDs in IMs, and their lesser improvement in positive symptoms compared to EMs may indicate the additional dopaminergic burden^32,51,52^, owing to the low activity of CYP2D6 in the brain.

This study had several limitations to be considered when interpreting the results. First, other polymorphisms affecting CYP2D6 enzyme activity, such as *41 or allele duplication, were not analyzed. However, since the frequencies of these polymorphisms are relatively low at <5% of the total polymorphisms^26^, they would not change the main results. Second, although APD-naive or -free patients with schizophrenia were recruited at the enrollment, part of the MRI data was acquired after the initiation of APD treatment. Since the neurobiological effect of APDs on WM connectivity is very complex^53,54^, future studies will be warranted to exclude the confounding effect of APDs. Third, the direction of causality remains unknown owing to the cross-sectional design of this study. A prospective and longitudinal or long-term outcome approach is required to verify the causal direction in the association among CYP2D6 variation, WM connectivity, and the neurobiology of schizophrenia.

In conclusion, this was the first study to demonstrate the genetic influence of CYP2D6 polymorphism on hippocampal WM connectivity and the treatment response after APD treatment. Decreased CYP2D6 activity may confer susceptibility to the development of schizophrenia by increasing hippocampal hyperactivity and dopaminergic burden. Furthermore, CYP26D-associated hippocampal WM alterations could be a possible endophenotype for schizophrenia that accounts for individual differences in clinical features and treatment response.

## Methods

### Participants

Patients with schizophrenia were recruited from the Department of Psychiatry, CHA Bundang Medical Center (Seongnam, Republic of Korea). All participants were of Korean descent and met the diagnostic criteria for schizophrenia from the Diagnostic and Statistical Manual of Mental Disorders, Fourth Edition, Text Revision (DSM-IV-TR). The diagnostic interview was administered by the experienced psychiatrists using the Structured Clinical Interview for DSM-IV-TR Axis I Disorders^55,56^. We only recruited participants with schizophrenia who were APD-naive or free of APDs at least for 6 months. Participants were excluded if they had any current or past history of (1) other psychiatric disorders, including mood disorders and substance-related disorders; (2) intellectual disability; (3) medical or neurological disorders or head trauma with loss of consciousness; and (4) any other contraindications for MRI scan. Handedness was assessed using the Edinburgh Handedness Inventory^57^ and left-handed participants were excluded. A total of 106 participants with schizophrenia were finally included in the present study.

All study procedures were reviewed and approved by the Institutional Review Board of CHA Bundang Medical Center, in accordance with the latest version of the Declaration of Helsinki and principles of Good Clinical Practice. All participants provided written informed consent following a thorough explanation of the study procedures.

### Clinical assessment

All participants with schizophrenia were assessed for the severity of their clinical symptoms using the PANSS^30^ at baseline. Sixty five participants (61.3%) were followed-up with for the assessment of treatment response after 4 weeks of APD treatment as recommended in standard practice. Treatment response was defined as the percentage of reduction in each subscale of the PANSS (positive symptom, negative symptom, and general psychopathology) over 4 weeks. Since the PANSS adopts a 1–7 scoring system, the percentage of reduction in each subscale of the PANSS could not be 100%, and it could be up to 85.7% (ref. ^58^).

### Genotyping

Genotyping was performed to analyze the CYP2D6*10 and CYP2D6*5 polymorphisms of the CYP2D6 gene. Briefly, the genomic DNA flanking the SNPs of interest was amplified using polymerase chain reaction (PCR) with forward and reverse primer pairs, and standard PCR reagents in 10 µL reaction volume, containing 10 ng of genomic DNA, 0.5 pM of each oligonucleotide primer, 1 µL of 10× PCR buffer, 250 μM dNTP (2.5 mM each), and 0.25 units of i-StarTaq DNA polymerase (5 units/µL). The PCR reactions were performed as follows: 1 cycle at 95 °C for 10 min; followed by 35 cycles at 95 °C for 30 s, 60 °C for 1 min, and 72 °C for 1 min; further followed by 1 cycle at 72 °C for 10 min. After amplification, the PCR products were treated with one unit each of shrimp alkaline phosphatase and exonuclease I at 37 °C for 75 min and 72 °C for 15 min to purify the amplified products. The PCR products were sequenced using the ABI PRISM SNaPShot Multiplex kit (Applied Biosystems; Foster City, CA, USA) and ABI PRISM 3730xl DNA analyzer (Applied Biosystems, Foster City; CA, USA). Primers of CYP2D6*10 used in this study were 5′-CATTTGGTAGTGAGGCAGGT (forward primer), 5′-TGGTCGAAGCAGTATGGTG (reverse primer), and 5′-GCGCCAACGCTGGGCTGCACGCTAC (genotyping primer). The CYP2D6*5 allele was detected using the long PCR, as described previously^59,60^.

The CYP2D6 alleles were classified as the phenotypes of EM, IM, PM, and UM according to the expected enzyme activities reported on the database of the Pharmacogene Variation Consortium (PharmVar; https://www.pharmvar.org/gene/CYP2D6). Based on the Clinical Pharmacogenetics Implementation Consortium guidelines for CYP2D6 genotype and codeine therapy^61^, AS values were assigned to each allele as follows: 0 for PM; 0.5–1.0 for IM; 1.5–2.0 for EM; and >2.0 for UM. The total CYP2D6 AS was calculated as the sum of the values assigned to each allele (CYP2D6*5 = 0, CYP2D6*10(T) = 0.5, CYP2D6*10(C) = 1.0 [major allele: C, minor allele: T]).

### MRI data acquisition

MRI data acquisition was performed at CHA Bundang Medical Center using a 3.0-Tesla GE Signa HDxt scanner (GE Healthcare, Milwaukee, WI, USA). Diffusion-weighted images (DWI) were acquired using an echo planar imaging (EPI) sequence with the following parameters: repetition time, 17,000 ms; echo time, 108 ms; field of view, 240 mm; matrix, 144 × 144; slice thickness, 1.7 mm; and voxel size, 1.67 × 1.67 × 1.7 mm^3^. A double-echo option was applied to reduce eddy-current-related distortions. To reduce the impact of EPI spatial distortions, an 8-channel coil and ASSET (Array of Spatial Sensitivity Encoding Techniques; GE Healthcare) with a SENSE factor of two was used. Seventy axial slices parallel to the anterior commissure–posterior commissure line were acquired in 51 directions with *b* = 900 s/mm^2^. Eight baseline scans with *b* = 0 s/mm^2^ were also acquired. DTIs were extracted from the DWIs using the least-squares method.

### DTI analysis

Analysis of DTI data was conducted using the Functional MRI of the Brain (FMRIB) Diffusion Toolbox and Tract-based Spatial Statistics (TBSS), implemented in the FMRIB Software Library (FSL version 6.0; Oxford, UK; https://fsl.fmrib.ox.ac.uk/fsl/). DTIs underwent standard preprocessing steps, including skull stripping and eddy-current correction. FA images were created by fitting a tensor model to the corrected diffusion data and then aligned in the Montreal Neurologic Institute standard space. Transformed FA images were combined and applied to the original FA map to produce a standard-space version of the FA map. A mean FA image, created by averaging all transformed FA images, was thinned to obtain a mean FA skeleton representing the centers of the WM tracts. The skeleton was thresholded using an FA > 0.2 to involve only major fiber bundles. Non-FA (MD, AD, and RD) images were prepared in a similar way as provided by TBSS.

Prior to conducting voxel-wise statistical analysis of DTI data, hippocampal WM regions^62,63^ (hippocampal cingulum, fornix, and stria terminalis) were chosen as ROIs based on the JHU DTI-based probabilistic tractography atlas^29^. Statistical analysis of DTI data was performed within the ROI mask using permutation-based nonparametric inference within the framework of a general linear model. There were 10000 permutations, and multiple comparisons were adjusted using the threshold-free cluster enhancement (TFCE) method. Analysis of covariance with sex, age, ICV, duration of illness, and APD dose at scan as covariates was additionally conducted to rule the confounding effects of these variables on the results. Significance was set at *p* < 0.05.

### Statistical analyses

To compare the sociodemographic and clinical characteristics, independent *t* tests for continuous variables and chi-squared tests for categorical variables were used. Correlation analyses were performed to confirm the associations among CYP2D6 activity, hippocampal WM connectivity, and treatment response. Statistical procedures were performed using SPSS version 26 (IBM Corporation; Armonk, NY, USA). Significance was set at *p* < 0.05.

### Reporting summary

Further information on research design is available in the Nature Research Reporting Summary linked to this article.

## Supplementary information

Reporting summary

## Data Availability

The data supporting the findings of this study are not publicly available due to ethical restrictions for protecting participants’ confidentiality and privacy, but are accessible from the corresponding author on reasonable request with the approval of the Institutional Review Board of CHA Bundang Medical Center.

## Code availability

No custom code or mathematical algorithm was used in this study.
